# Cytoprotective Effect of *Bambusae caulis* in Liquamen by Blocking Oxidative Stress in Hepatocytes

**DOI:** 10.3390/molecules28155862

**Published:** 2023-08-03

**Authors:** Ji Hye Yang

**Affiliations:** College of Korean Medicine, Dongshin University, Naju 58245, Republic of Korea; uranus2k@nate.com; Tel.: +82-61-330-3518

**Keywords:** *Bambusae caulis* in Liquamen, therapeutic candidate, tert-butyl hydroperoxide, traditional herbal medicine, oxidative stress, hepatocyte damage

## Abstract

*Bambusae caulis* in Liquamen (BCL), which is extracted from heat-treated fresh bamboo stems, is a traditional herbal medicine widely used in Eastern countries. Recently, it has been reported to have anti-inflammatory and whitening effects. However, the protective effect of BCL on hepatocytes has not yet been elucidated. The present study aimed to determine whether BCL prevents oxidative stress induced by tert-butyl hydroperoxide (t-BHP) and exerts cytoprotective effects on hepatocytes. High-performance liquid chromatography and liquid chromatography with tandem mass spectroscopy were performed to analyze the type of polyphenols present in BCL. The activities of antioxidant enzymes and hepatocyte viability were assessed. The benzoic acid content was the highest among polyphenols present in BCL. Benzoic acid acts as a scavenger of free radicals, including reactive oxygen species. BCL increased the expression of antioxidant enzymes (glutamate–cysteine ligase and NADPH quinone dehydrogenase (1)) by activating nuclear factor erythroid 2-related factor 2 and reduced tBHP-induced cell death by inhibiting oxidative stress. BCL inhibited tBHP-induced phosphorylation of p38 and c-Jun N-terminal kinase but not that of extracellular signal-regulated kinase. In conclusion, BCL is a promising therapeutic candidate for treating oxidative-stress-induced hepatocyte damage.

## 1. Introduction

Liver diseases encompass a broad range of conditions that affect the liver and can lead to the death of hepatocytes, the main functional cells of the liver. Hepatocytes play a crucial role in numerous vital functions, including detoxification, protein synthesis, metabolism, and bile production [1]. Therefore, hepatocyte damage or death has significant implications for liver health. In liver diseases, hepatocyte death can occur via the apoptotic pathway, also known as programmed cell death [2]. Excessive reactive oxygen species (ROS) can modulate the activity of proapoptotic proteins, such as the B-cell lymphoma 2 (Bcl-2) family members (Bcl-2-associated X protein (Bax), Bcl-2 associated agonist of cell death (Bad), and B-cell lymphoma extra large (Bcl-xL)) and caspase 3 [3]. The interplay between the Bcl-2 family members and caspase 3 is crucial for the regulation and execution of apoptosis. The balance between antiapoptotic and proapoptotic Bcl-2 family members determines the susceptibility of a cell to apoptosis, while the activation of caspase 3 serves as the final step in executing cell death [4,5].

Cells have a defense system that includes antioxidant enzymes against ROS. These enzymes neutralize and scavenge ROS, preventing them from harming cellular components. The key factor for regulating the expression of antioxidant enzymes is nuclear factor erythroid 2-related factor 2 (Nrf2). Under normal conditions, Nrf2 is sequestered in the cytoplasm by Kelch-like ECH-associated protein 1 (Keap1). Keap1 facilitates the degradation of Nrf2, preventing its translocation into the nucleus. However, in response to oxidative stress or electrophilic insult, Nrf2 is released from Keap1 and translocated to the nucleus [6,7]. Once in the nucleus, Nrf2 binds to the antioxidant response element (ARE), a specific DNA sequence present in the promoter regions of genes encoding various antioxidant and detoxification enzymes [8]. ARE acts as a regulatory element for the genes encoding antioxidant enzymes; therefore, strategies aimed at enhancing Nrf2 activation have been explored as potential therapeutic interventions for treating oxidative-stress-associated diseases [9,10].Tert-butyl hydroperoxide (tBHP) is an organic peroxide that is commonly used as a radical initiator or oxidizing agent in various chemical reactions [11]. Owing to its high reactivity and ability to release free radicals, tBHP is valuable for processes such as polymerization and oxidation reactions [11].

Bamboo is a grass belonging to the Poaceae family, known for its rapid growth and versatility. Bamboo stems, also known as culms, are the main structural components of bamboo [12]. *Bambusae caulis* in Liquamen (BCL), which is extracted from heat-treated fresh bamboo stems, is a traditional herbal medicine widely used in Eastern countries for treating cough and asthma [13]. Recently, research on the efficacy of BCL has been increasing; Qi et al. have reported the whitening effect of BCL, and Park et al. have confirmed the anti-inflammatory efficacy of BCL [14,15]. However, the hepatic protective effects of BCL have not yet been elucidated. Therefore, this study aimed to determine whether BCL prevents tBHP-induced oxidative stress and exerts cytoprotective effects on hepatocytes.

## 2. Results

### 2.1. Antioxidative Potential and Component Analysis of BCL

**Figure 1:**
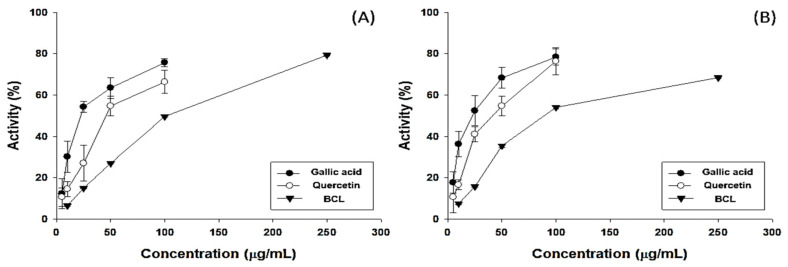
Antioxidative potential of *Bambusae caulis* in Liquamen (BCL). Free-radical-scavenging assays using (**A**) 2,2-diphenylpicrylhydrazil (DPPH) and (**B**) 2,2′-azino-bis (3-ethylbenzothiazoline-6-sulfonic acid (ABTS) of gallic acid, quercetin, and BCL. Experiments were performed in triplicate and repeated three times with similar results.

**Figure 2:**
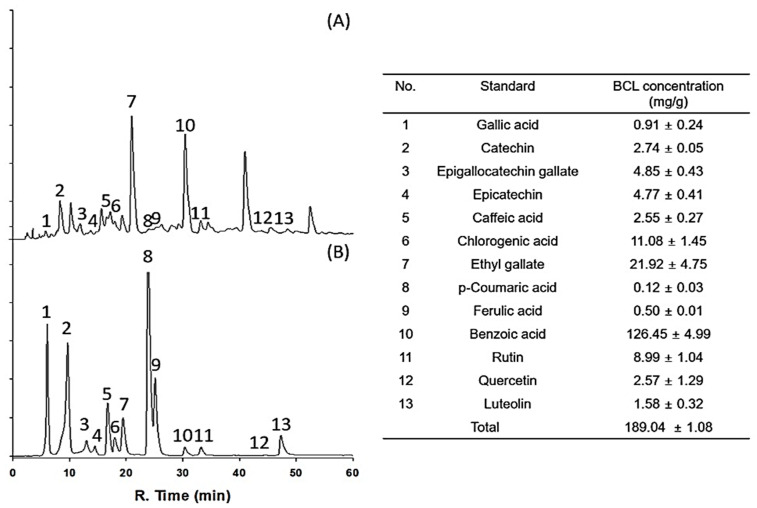
High-performance liquid chromatogram of *Bambusae caulis* in Liquamen (BCL) (**A**) and standard mixture (**B**) using diode array detection at 280 nm. Numbers indicate the following: (1) gallic acid, (2) catechin, (3) epigallocatechin gallate, (4) epicatechin, (5) caffeic acid, (6) chlorogenic acid, (7) ethyl gallate, (8) p-coumaric acid, (9) ferulic acid, (10) benzoic acid, (11) rutin, (12) quercetin, and (13) luteolin.

The antioxidant potential of fractionated BCL was measured using 2,2-diphenyl-1-picrylhydrazyl (DPPH) and 2,2′-azinobis-(3-ethylbenzothiazoline-6-sulfonic acid) (ABTS) radical-scavenging assays [16]. The half-maximal inhibitory concentration (IC_50_) of BCL was 99.97 ± 1.23 and 88.17 ± 0.82 μg/mL in the DPPH and ABTS radical-scavenging assays, respectively (Figure 1). The total polyphenol content (TPC) and total flavonoid content (TFC) of BCL were determined using colorimetric methods. BCL showed TPC and TFC values of 274.13 ± 15.49 gallic acid equivalent (GAE) mg/g and 352.27 ± 1.28 quercetin equivalent mg/g, respectively (Table 1). High-performance liquid chromatography (HPLC) analysis was performed to analyze the type of polyphenols present in BCL. Thirteen phenolic acid and flavonoid standards (gallic acid, catechin, epigallocatechin gallate, epicatechin, caffeic acid, chlorogenic acid, ethyl gallate, p-coumaric acid, ferulic acid, benzoic acid, rutin, quercetin, and luteolin) were selected to analyze BCL (Figure 2). Benzoic acid was the major component, followed by ethyl gallate and chlorogenic acid. Liquid chromatography–tandem mass spectroscopy (LC-MS/MS) analysis was performed to verify the results of the HPLC analysis. Consistently, the benzoic acid content was the highest at 23.14 mg/g (Table 2).

### 2.2. BCL-Induced Antioxidant Enzymes and Nrf2 Activation in Hepatocytes

**Figure 3:**
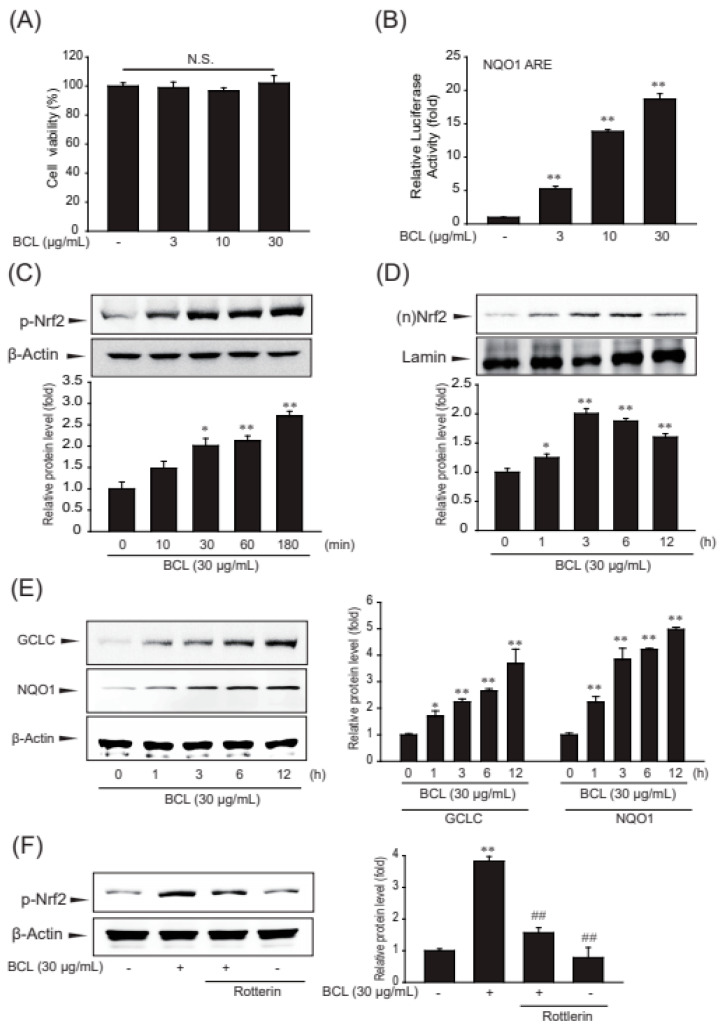
Effect of BCL on the expression of antioxidant enzymes and nuclear factor erythroid 2 related factor 2 (Nrf2) activation in HepG2 cells. (**A**) The effect of *Bambusae caulis* in Liquamen (BCL) (1–30 μg/mL, 24 h) on cytotoxicity was estimated using MTT assay in HepG2 cells. Experiments were repeated three times. N.S.: not significant. (**B**) BCL-induced luciferase activity of NQO1-ARE was measured in the lysates of HepG2 cells transfected with the NQO1-ARE luciferase construct. Experiments were repeated three times. (**C**) Phosphorylation of Nrf2 was measured by immunoblot analysis of lysates of HepG2 cells incubated with 30 μg/mL of BCL for 30 min to 6 h. Experiments were repeated three times. (**D**) The time-course study of nuclear translocation of Nrf2 in HepG2 cells treated with BCL (30 μg/mL) for 1–12 h. Experiments were repeated thrice. (**E**) Effect of BCL treatment on the expression of antioxidant enzymes, such as glutamate-cysteine ligase catalytic subunit (GCLC) and NADPH quinone dehydrogenase 1 (NQO1) in HepG2 cells. Cells were stimulated with BCL (30 μg/mL) for 1–12 h. (**F**) Effects of PKC-δ on Nrf2 phosphorylation activity in BCL-treated cells. Cells were pretreated with rottlerin (a PKC-δ inhibitor, 25 μM) for 1 h and stimulated with BCL (30 μg/mL) for 3 h. Data represent mean ± standard error (S.E.) of three experiments; * *p* < 0.05 and ** *p* < 0.01, vs. vehicle-treated control; ## *p* < 0.01 vs. BCL treatment alone.

The assay using 3-(4,5-dimethylthiazol-2-yl)-2,5-diphenlyltetrazolium bromide (MTT) showed that BCL up to 30 μg/mL concentration was not cytotoxic for HepG2 cells (Figure 3A). Nrf2-target genes contain AREs in the promoter region. AREs were initially identified as cis-regulatory elements for NADPH quinone dehydrogenase 1 (NQO1) and glutathione S-transferase (GST) genes [17,18]. To examine the effect of BCL on Nrf2 activity, the luciferase activity of NQO1-ARE following treatment with BCL was measured. BCL increased the luciferase activity of NQO1-ARE (Figure 3B). Moreover, the effects of BCL on increasing the phosphorylation and nuclear translocation of Nrf2 were investigated. The nuclear translocation and activation of Nrf2 are modulated by post-translational modifications, mainly phosphorylation. Phosphorylated Nrf2 translocates to the nucleus and increases the expression of its target genes [19]. Treatment with BCL gradually increased the Nrf2 phosphorylation from 10 min to 3 h. The nuclear translocation of Nrf2 peaked at 3 h of treatment with BCL and then decreased (Figure 3C,D).

The genes of glutamate–cysteine ligase (GCL) and NQO1 are well-known targets of Nrf2, and these enzymes protect cells from oxidative stress. As expected, the expression of antioxidant enzymes, such as GCL and NQO1, was increased by treatment with BCL in a time-dependent manner (Figure 3E). PKC-δ is the major PKC isoform that phosphorylates Nrf2 Ser40. Therefore, rottlerin, a PKCδ inhibitor, was used to determine whether BCL activates the Nrf2 pathway through PKC-δ-mediated phosphorylation. Therefore, BCL induced Nrf2-mediated GCL and NQO1 expression through PKC-δ in HepG2 cells (Figure 3F).

### 2.3. BCL Suppressed Oxidative Stress and Cell Death Induced by t-BHP

**Figure 4:**
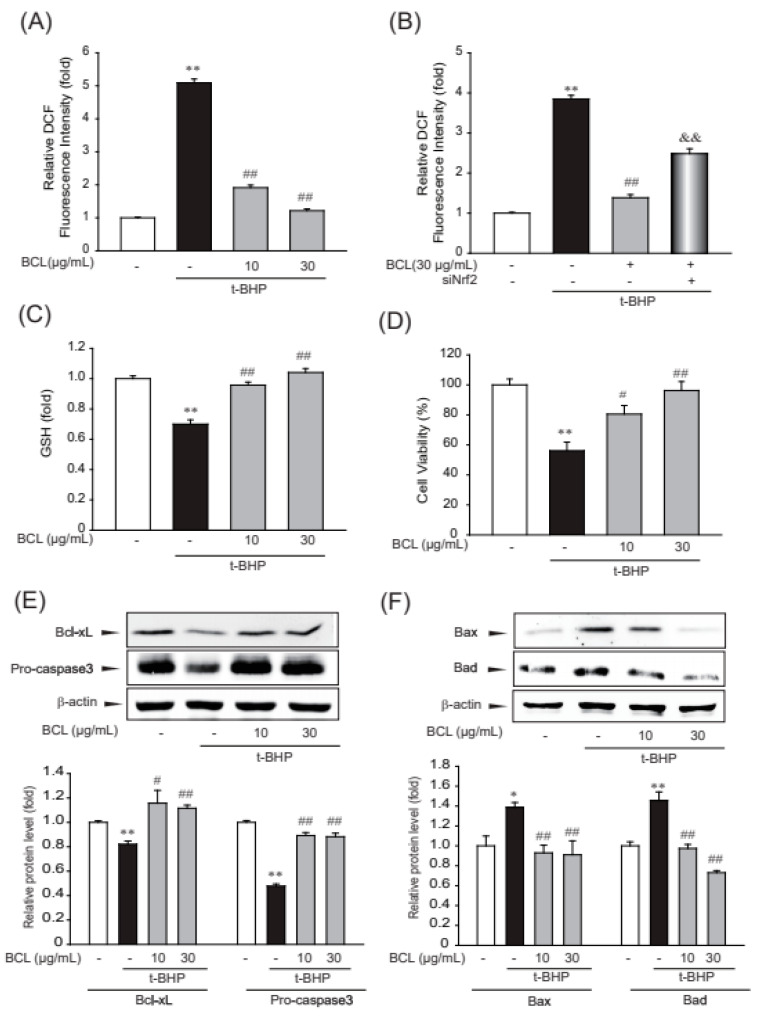
The cytoprotective efficacy of BCL in HepG2 cells. (**A**,**B**) The efficacy of *Bambusae caulis* in Liquamen (BCL) on tert-butyl hydroperoxide (tBHP)-induced production of reactive oxygen species (ROS). HepG2 cells were pretreated with 10–30 μg/mL BCL for 1 h and then incubated with 500 μM tBHP for 1 h (**A**). Antioxidant effects of BCL, mediated by the activation of the Nrf2 pathway, were assessed using siNrf2. After siNrf2 transfection for 48 h, cells were pretreated with BCL 30 μg/mL and then incubated with 500 μM tBHP for 1 h (**B**). Cells were stained with 10 μM diacetyldichlorofluorescein (DCFH-DA) at 37 °C for 30 min. Intracellular fluorescence intensities were measured using a fluorescence microplate reader. The experiments were repeated thrice. (**C**) Reduced glutathione (GSH) levels were measured in the lysates of cells that were treated with tBHP (500 μM) and/or 10–30 μg/mL BCL for 2 h. The experiments were repeated thrice. (**D**) The efficacy of BCL on tBHP-induced cell death. HepG2 cells were treated with tBHP (500 μM) and/or 10–30 μg/mL BCL for 3 h. Cell viability was assessed using an MTT assay. The experiments were repeated thrice. (**E**,**F**) Representative Western blots analyzing the levels of proteins involved in cell death in the presence or absence of BCL and/or tBHP. The levels of marker proteins (Bax, Bad, Bcl-xL, and procaspase 3) of apoptosis in HepG2 cell lysates as determined by immunoblotting. The experiments were repeated thrice. Data represent the mean ± standard error (S.E.) of three experiments; * *p* < 0.05 and ** *p* < 0.01 vs. vehicle-treated control; # *p* < 0.05 and ## *p* < 0.01 vs. tBHP treatment alone; && *p* < 0.01 vs. t-BHP and BCL treatment.

To determine the inhibitory effects of BCL on oxidative stress, ROS production and intracellular levels of reduced glutathione (GSH) were measured [20]. BCL inhibited tBHP-induced ROS production via Nrf2 and maintained the level of intracellular GSH that was reduced by treatment with tBHP (Figure 4A–C). Oxidative stress caused by ROS production plays a major role in cellular apoptosis [21]. To investigate whether BCL can reduce cellular apoptosis, cell viability was determined using the MTT assay and analyzing the expression of inducible proteins (Bax, Bad, Bcl-xL, and procaspase 3) that are markers of the canonical apoptosis pathway. Treatment with BCL reduced tBHP-induced cell death and the expression of Bax and Bad in a dose-dependent manner (Figure 4C,D). Moreover, BCL recovered the expression of Bcl-xL and procaspase 3 (Figure 4E). These results indicate that BCL can reduce cell death by inhibiting tBHP-induced oxidative stress in HepG2 cells.

### 2.4. Effect of BCL on p38 and c-Jun N-terminal Kinase (JNK) Phosphorylation

**Figure 5:**
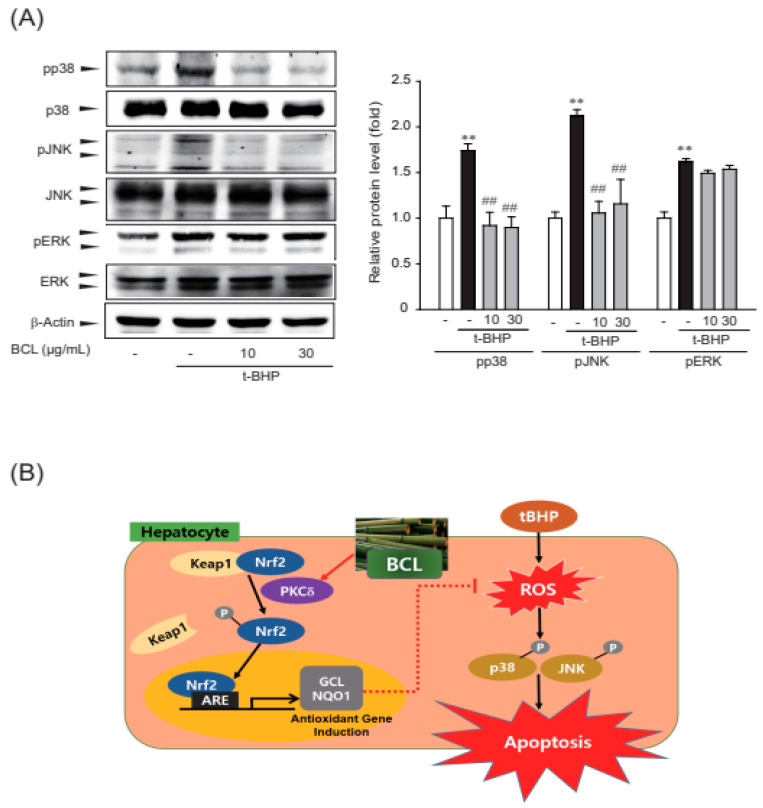
Inhibition of p38 and c-Jun N-terminal kinase (JNK) phosphorylation by *Bambusae caulis* in Liquamen (BCL) in tBHP-treated HepG2 cells. (**A**) Effect of BCL on tBHP-induced phosphorylation of MAPKs. Cells were treated with BCL for 1 h before tBHP stimulation for 30 min. Cell lysates were immunoblotted, and results were confirmed by repeated experiments. Data represent mean ± S.E. of three experiments; ** *p* < 0.01 vs. vehicle-treated control; ## *p* < 0.01 vs. tBHP treatment alone. (**B**) Schematic diagram illustrating the mechanism of cytoprotective effect of BCL in HepG2 cells.

ROS are produced during the stress response, acting upstream or downstream of mitogen-activated protein kinase (MAPK), including extracellular signal-regulated kinase (ERK), p38, and JNK [22,23]. Therefore, the effect of BCL on tBHP-induced phosphorylation of MAPKs was assessed. BCL inhibited tBHP-induced phosphorylation of p38 and JNK but not that of ERK (Figure 5A). Therefore, BCL may inhibit p38 and JNK phosphorylation to meditate its cell protection effects.

## 3. Discussion

The traditional herbal medicine derived from heat-treated stems of bamboo, BCL, has various effects, such as cooling and diuretic, expectorant, and digestive properties [24]. It possesses immuno-regulatory properties that can suppress the expression of thymus and activation-regulated chemokines and macrophage-derived factors [14]. In addition, it exerts whitening effects by inhibiting tyrosinase activity and melanin production in B16F10 melanoma [15]. However, the role of BCL in oxidative-stress-induced hepatocyte damage has not been elucidated. Our study indicated that BCL prevents tBHP-induced oxidative stress and exerts cytoprotective effects on hepatocytes. In the present study, high amounts of benzoic acid were observed in BCL. Benzoic acid is a common food preservative and is naturally found in various fruits and berries [25]. It has antimicrobial properties and is used to inhibit the growth of bacteria, yeasts, and molds in food and cosmetic products [26,27]. Several studies have investigated the antioxidant properties of benzoic acid; in vitro experiments using cultured cells have suggested that benzoic acid exerts antioxidant activity by reducing ROS levels and preventing oxidative damage [28,29]. However, such studies are limited, and the exact mechanisms and extent of antioxidant effects exerted by benzoic acid are not fully understood yet.

In the present study, BCL induced the expression of antioxidant enzymes and suppressed oxidative stress and cell death caused by t-BHP in hepatocytes. Cells have a defense system against the harmful effects of excessive ROS, including antioxidant enzymes NQO1 and GCLC. NQO1 catalyzes the reduction of quinones and other oxidized compounds using NAD(P)H and prevents the generation of highly reactive and potentially damaging ROS [30]. GCLC is a key regulator of GSH, which is crucial for cellular defense against ROS as it acts as a reducing agent and can directly scavenge ROS, such as H_2_O_2_. Moreover, GCLC catalyzes the rate-limiting step of GSH synthesis and is important for maintaining optimal GSH levels [31]. These enzymes are crucial for maintaining cellular redox homeostasis and protecting cells from oxidative stress-induced damage.

In the present study, treatment with BCL upregulated the expression of Nrf2 target genes, such as GCLC and NQO1, to protect cells from ROS induced by tBHP. Moreover, the activation of the Nrf2 pathway by BCL was confirmed to be mediated through protein kinase Cδ (PKCδ). Nrf2 phosphorylation at the Ser40 residue, mediated by protein kinase Cδ (PKCδ), disrupts Nrf2 and Keap1 association, promoting the translocation of Nrf2 to the nucleus [32]. We also conducted experiments using siNrf2 to show the antioxidant effect of BCL through activation of the Nrf2 pathway. Treatment with BCL reduced tBHP-induced ROS, whereas siNrf2 cells do not.

In our study, BCL inhibited cell death induced by tBHP by blocking the phosphorylation of p38 and JNK. ROS and the MAPK signaling pathways are interconnected and associated with cell death. The ROS-mediated activation of MAPKs can contribute to cell death by modulating the expression of proapoptotic proteins, promoting mitochondrial dysfunction, or initiating apoptotic cascades [33,34,35]. Therefore, BCL, which induce Nrf2-mediated antioxidant enzymes, can reduce tBHP-induced ROS. In addition, it prevents cell death via blocking p38 and JNK phosphorylation in hepatocytes.

In future studies, the hepatoprotective effects of BCL should be investigated using animal models of liver disease.

## 4. Materials and Methods

### 4.1. Preparation of BCL

**Figure 6:**
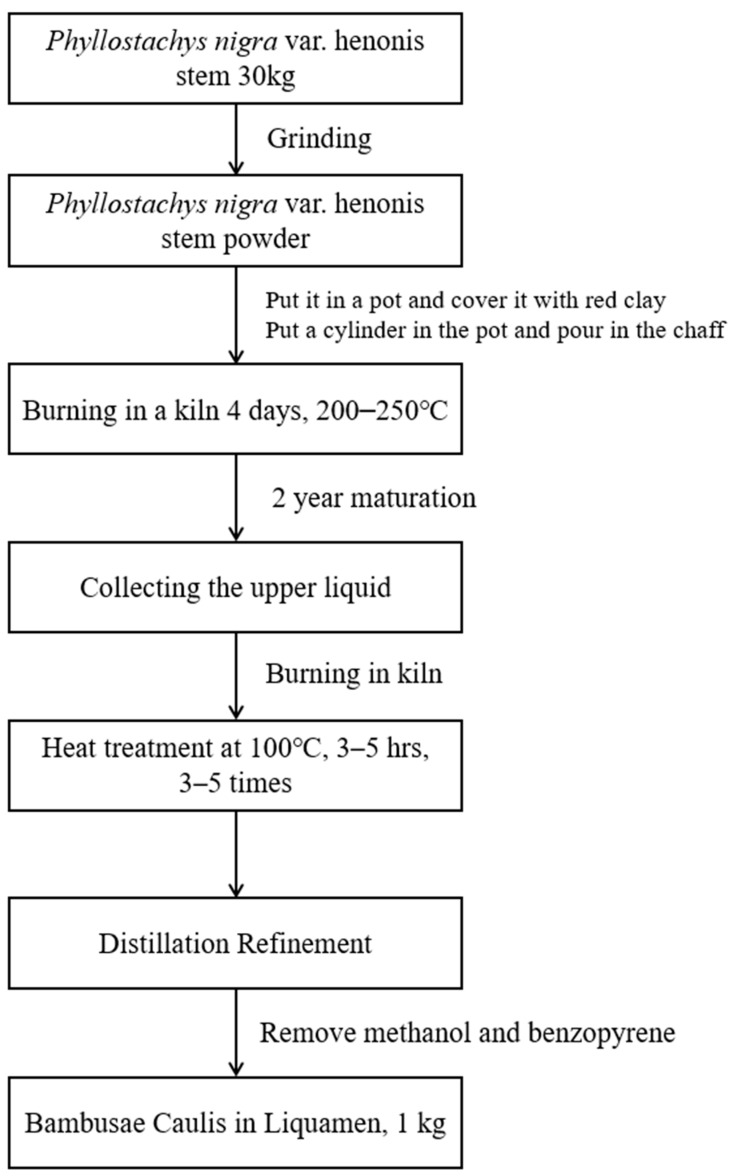
Schematic diagram of *Bambusae Caulis* in Liquamen (BCL) preparation.

A total of 18 L of BCL purchased from Bamboo Forest Foods Co., Ltd. (648 Samdari, Damyang-eup, Damyang-gun, Jeollanam-do, Republic of Korea) was concentrated using a decompression concentrator. The detailed procedure for preparing BCL is shown in Figure 6. The concentrated solution was used for further analyses.

### 4.2. Materials

Antibodies against Nrf2, phospho-Nrf2, Bax, and Bcl-xL were purchased from Santa Cruz Biotechnology (Santa Cruz, CA, USA). NQO1, lamin A/C, and caspase-3 antibodies were obtained from Cell Signaling (Danvers, MA, USA). Glutamate cysteine ligase (GCL) antibody was purchased from Abcam (Cambridge, UK) and a Bad antibody was purchased from BD biosciences (Franklin Lakes, NJ, USA). MTT, metaphosphoric acid, DCFH-DA, t-butylhydroperoxide (t-BHP), rotenone, dimethylsulfoxide, and β-actin antibody were purchased from Sigma-Aldrich (St. Louis, MO, USA). siNrf2 was purchased from Dharmacon (Lafayette, CO, USA).

### 4.3. Radical Scavenging Activity Assay Using DPPH

To a 200 μL sample of bamboo vinegar (BCL), 800 μL of 0.25 mM DPPH reagent was added, and the mixture was allowed to react in the dark for 15 min. The absorbance at 517 nm was measured using a UV-Vis spectrophotometer (SCINCO, Seoul, Republic of Korea).

Radical scavenging activity (%) = (Abs_control_ − Abs_sample_)/Abs_control_ × 100

### 4.4. Radical Scavenging Activity Assay Using ABTS

A 7 mM solution of ABTS was mixed with 2.45 mM potassium persulfate, and the resulting mixture was allowed to react in the dark at room temperature for 12 h to generate ABTS radical cation. The solution containing ABTS radical cation was then diluted with phosphate-buffered saline (pH 7.4) to an absorbance value of 0.80 ± 0.02. Next, 200 μL of the sample with concentrations ranging from 10 to 1000 μg/mL was mixed with 1000 μL of diluted ABTS radical solution. The mixture was incubated in the dark for 15 min, and the absorbance at 720 nm was measured using a SCINCO UV-Vis spectrophotometer s-3100 (Seoul, Republic of Korea).

Radical scavenging activity (%) = (Abs_control_ − Abs_sample_)/Abs_control_ × 100

### 4.5. Total Phenolic Content

Bamboo vinegar (500 μg/mL) was mixed with 0.2 M Folin–Ciocalteu solution and 2% Na_2_CO_3_ (*w*/*v*) at a 1:1:1 ratio. After allowing the mixture to react for 30 min at room temperature, absorbance at 750 nm was measured using a UV-Vis spectrophotometer (SCINCO).

### 4.6. Total Flavonoid Content

A total of 1.5 mL of methanol, 100 μL of 10% aluminum chloride, 100 μL of 1 M potassium acetate, and 2.8 mL distilled water were added to 500 μL of a sample containing 500 μg/mL bamboo vinegar, and the mixture was allowed to react at room temperature for 40 min. Subsequently, absorbance at 415 nm was measured using a UV-Vis spectrophotometer (SCINCO). Total flavonoid content was determined by constructing a calibration curve using quercetin as the standard.

### 4.7. Phenolic Compounds Identified in BCL Quantified by HPLC-MS/MS

HPLC–MS/MS analysis was performed using an AB SCIEX 4000 Q Trap LC/MS/MS System (Shimadzu LC 20A System; Kyoto, Japan). The mobile phases were water (0.1% formic acid, solvent (A)) and acetonitrile (0.1% formic acid, solvent (B)) under isocratic conditions (35% B). The analytical conditions for MS/MS were examined in both negative and positive modes using a Turbo Ion Spray. These data were obtained through the company SUMSUMBIO. Co., Ltd. (305, 123, Nanosandan-ro, Nam-myeon, Jangseong-gun, Jeollanam-do, Republic of Korea), an analysis company.

### 4.8. HPLC with Diode-Array Detection (HPLC–DAD) Analysis

The BCL was analyzed quantitatively using HPLC-DAD (SPD-20A, Shimadzu Co., Japan). Thirteen standards ((1) gallic acid, (2) catechin, (3) epigallocatechin gallate, (4) epicatechin, (5) caffeic acid, (6) chlorogenic acid, (7) ethyl gallate, (8) p-coumaric acid, (9) ferulic acid, (10) benzoic acid, (11) rutin, (12) quercetin, and (13) luteolin) were selected for experiments, and HPLC analysis conditions were as follows: Shim-pack GIS-ODS column (C18, 4.6 × 250 mm, 5.0 μm, Shimadzu Co.), flow rate of 0.7 mL/min, temperature 30 °C, injection volume 20 μL, and a UV detector wavelength of 280 nm. For the mobile phase, 0.1% acetic acid in water (solvent A) and 0.1% acetic acid in methanol (solvent B) were used. The gradient conditions of the mobile phase were 0 min: B (10%), 0–5 min: B (10%), 5–15 min: B (40%), 15–45 min: B (60%), 45–55 min: B (80%), 55–60 min: B (100%), 60–65 min: B (10%), 65–70 min: B (10%). The injection volume was 20 μL. All samples used for analysis were filtered with a 0.45 µm filter. These data were obtained through the company SUMSUMBIO. Co., Ltd.

### 4.9. Cell Culture

HepG2 cells obtained from American Type Culture Collection (Manassas, VA, USA) were maintained in Dulbecco’s modified Eagle’s medium containing 50 U/mL penicillin/streptomycin with 10% fetal bovine serum at 37 °C under 5% CO_2_ in a humidified atmosphere.

### 4.10. Cytotoxicity Assay

To measure cell viability, cells were plated in 48-well plates and treated for 24 h. Viable cells were then stained using 0.2 mg/mL MTT for 4 h as previously reported. The media were then removed and formazan crystals produced in the wells were dissolved using 200 μL of dimethyl sulfoxide. Absorbance at 540 nm was measured using a microplate reader (Spectramax; Molecular Devices, Sunnyvale, CA, USA). Cell viability was defined relative to the untreated control as:

Viability (% control) = 100 × (absorbance of treated sample)/(absorbance of control)

### 4.11. Measurement of ROS Generation

Diacetyldichlorofluorescein (DCFH-DA) is a cell-permeable and nonfluorescent probe that is cleaved by intracellular esterases and converted into highly fluorescent dichlorofluorescein upon reaction with H_2_O_2_. After treatment with 500 µM tert-butyl hydroperoxide (*t*-BHP) for 3 h, HepG2 cells were stained with 10 µM DCFH-DA for 30 min at 37 °C. H_2_O_2_ generation was determined by measuring the fluorescence intensity of dichlorofluorescein using a fluorescence microscope (Zeiss, Jena, Germany) or a fluorescence microplate reader (Jemini; Molecular Devices) using excitation and emission wavelengths of 485 and 530 nm, respectively.

### 4.12. Immunoblot Analysis

Protein extraction, subcellular fractionation, sodium dodecyl sulfate–polyacrylamide gel electrophoresis, and immunoblotting were performed as previously described [36]. Briefly, the samples were separated using 7.5% polyacrylamide gel and transferred to a nitrocellulose membrane. The membrane was incubated with the indicated primary antibodies, followed by incubation with horseradish peroxidase-conjugated secondary antibodies. Immunoreactive proteins were visualized using an ECL chemiluminescence detection kit (Amersham Biosciences, Buckinghamshire, UK). Equal loading of proteins and the integrity of subcellular fractionation were verified by β-actin expression in immunoblots.

### 4.13. Luciferase Assay

The NAD(P)H dehydrogenase (quinone) 1 (NQO1)-ARE luciferase construct, containing a 3-tandem repeat of the ARE in the 5′-upstream region of NQO1, was introduced into the cells to examine the transcriptional activation of Nrf2 by BCL. Briefly, after NQO1-ARE cells were replated in 12-well plates overnight, the cells were serum-starved for 6 h and treated with BCL for 12 h. The activity of firefly luciferase was then measured by adding Luciferase Assay Reagent II (Promega, Madison, WI, USA) according to the manufacturer’s instructions, and the Renilla luciferase reaction was initiated by adding Stop & Glo reagent (Promega). Relative luciferase activities were calculated by normalizing firefly luciferase activity to that of Renilla luciferase.

### 4.14. Statistical Analysis

One-way analysis of variance (ANOVA) was used to assess the statistical significance of differences among groups. For each statistically significant effect of treatment, the Newman–Keuls test was used for comparisons between multiple groups. The data were expressed as means ± standard deviation (SD) or standard error (S.E.).

## 5. Conclusions

Our result showed that BCL increased the expression of antioxidant enzymes, such as GCL and NQO1, and can reduce cell death by inhibiting tBHP-induced oxidative stress in hepatocytes. In addition, BCL prevented tBHP-induced phosphorylation of p38 and JNK. In conclusion, BCL exhibits antioxidative effects and can suppress cell death by blocking the phosphorylation of p38 and JNK. This study suggests BCL as a promising therapeutic candidate for treating oxidative-stress-induced hepatocellular damage.

## Figures and Tables

**Table 1:** Antioxidant potential (presented using the half-maximal inhibitory concentration (IC_50_)), total polyphenol content (TPC), and total flavonoid content (TFC) of *Bambusae caulis* in Liquamen (BCL).

Sample	DPPH (IC_50_ μg/mL)	ABTS (IC_50_ μg/mL)	TPC	TFC
			GAE mg/g	QUE mg/g
BCL	99.97 ± 1.23	88.17 ± 0.82	274.13 ± 15.49	352.27 ± 1.28
Gallic acid	22.30 ± 2.25	22.55 ± 1.12	-	-
Quercetin	46.21 ± 1.72	41.97 ± 1.89	-	-

GAE, gallic acid equivalent; QUE, quercetin equivalent.

**Table 2:** Component analysis of *Bambusae caulis* in Liquamen (BCL) using high-performance liquid chromatography and tandem mass spectroscopy.

No.	Standard	BCL Concentration (mg/g)
1	4-Hydroxy benzoic acid	0.10
2	Syringic acid	0.50
3	Coumaric acid	0.33
4	Benzoic acid	23.14
5	Nicotinic acid	0.14
6	Protocatechuic acid	0.08
7	Biochanin A	0.01
8	Catechin	0.08
9	Ethyl gallate	0.04
10	Epigallocatechin gallate	0.04
Total		24.47

## Data Availability

The data presented in this study are available in the article.
